# Towards the Prediction of Rearrest during Out-of-Hospital Cardiac Arrest

**DOI:** 10.3390/e22070758

**Published:** 2020-07-09

**Authors:** Andoni Elola, Elisabete Aramendi, Enrique Rueda, Unai Irusta, Henry Wang, Ahamed Idris

**Affiliations:** 1 Department of Communications Engineering, University of the Basque Country, 48013 Bilbao, Spain; elisabete.aramendi@ehu.eus (E.A.); enrique.rueda@ehu.eus (E.R.); unai.irusta@ehu.eus (U.I.); 2 Department of Emergency Medicine, University of Texas Health Science Center, Houston, TX 77030, USA; henry.e.wang@uth.tmc.edu; 3 Department of Emergency Medicine, University of Texas Southwestern Medical Center, Dallas, TX 75390, USA; ahamed.idris@utsouthwestern.edu

**Keywords:** out-of-hospital cardiac arrest (OHCA), rearrest, electrocardiogram (ECG), heart rate variability (HRV), random forest (RF)

## Abstract

A secondary arrest is frequent in patients that recover spontaneous circulation after an out-of-hospital cardiac arrest (OHCA). Rearrest events are associated to worse patient outcomes, but little is known on the heart dynamics that lead to rearrest. The prediction of rearrest could help improve OHCA patient outcomes. The aim of this study was to develop a machine learning model to predict rearrest. A random forest classifier based on 21 heart rate variability (HRV) and electrocardiogram (ECG) features was designed. An analysis interval of 2 min after recovery of spontaneous circulation was used to compute the features. The model was trained and tested using a repeated cross-validation procedure, on a cohort of 162 OHCA patients (55 with rearrest). The median (interquartile range) sensitivity (rearrest) and specificity (no-rearrest) of the model were 67.3% (9.1%) and 67.3% (10.3%), respectively, with median areas under the receiver operating characteristics and the precision–recall curves of 0.69 and 0.53, respectively. This is the first machine learning model to predict rearrest, and would provide clinically valuable information to the clinician in an automated way.

## 1. Introduction

Cardiac arrest remains a major public health problem with more than 275,000 out-of-hospital cardiac arrest (OHCA) cases treated yearly in Europe [1], and survival rates below 10% [2,3]. Prompt treatment is crucial because the probability of survival decreases by 10% for every minute treatment is delayed [4,5]. Current cardiopulmonary resuscitation (CPR) guidelines define chain of survival to ensure a prompt OHCA treatment, with five important links [6]: early recognition of the arrest, CPR with chest compressions and ventilations, rapid defibrillation, basic/advanced emergency medical treatment, and post-cardiac arrest care.

The final aim of the treatment provided by the emergency medical services is to achieve the return of spontaneous circulation (ROSC), and to then proceed to the last link of the chain of survival, post-arrest treatment, and transportation to hospital. CPR manoeuvres, defibrillation, and drugs produce changes in the patient’s state, which are reflected in the cardiac rhythm. For instance, defibrillation may bring a patient from ventricular fibrillation to a rhythm with spontaneous pulse, that is, to ROSC.

Rearrest is experienced by patients who achieve ROSC and suffer a subsequent cardiac arrest during their prehospital care. Rearrest is frequent in the prehospital setting with observed incidences between 24% and 43% [7,8,9,10]. Moreover, rearrest is associated to poorer patient outcomes, both for hospital discharge and neurological state at follow up [7,8,9,10,11]. The prediction of rearrest would contribute to better outcomes by providing adequate medical treatment to better stabilize the patient, and by delaying transport to hospital, as providing adequate care is more difficult when rearrest occurs in an ambulance during transport to hospital.

Several characteristics observable in the electrocardiogram (ECG) are associated to rearrest risk factors: low heart rate, increased heart rate variability, long QRS complexes, irregular beats, etc. Nevertheless, very few automated methods have been proposed to predict rearrest. Some important contributions by Salcido et al. in the use of heart rate variability (HRV) features and morphology features of the ECG [9,12] showed the potential of the ECG in this context. Other studies focused on the transition between cardiac rhythms, including the transition from pulse-generating rhythms (ROSC) to non-pulsatile rhythms, that is, rearrest [13].

In this paper, a machine learning technique is developed to predict rearrest in OHCA patients. A solution based on a random forest (RF) classifier is adjusted for 1 and 2 min of ECG signal acquired by the defibrillation pads, a signal commonly recorded by all defibrillators in OHCA scenarios. In the Materials section, the source of the OHCA cases and the ECG signals is described. The HRV features and the design of the RF classifier is detailed in Methods, and the Results are given next. In the Discussion section, the clinical importance and implications in OHCA treatment of this algorithm are elaborated.

## 2. Data Collection

The data used in this study were a subset of a large OHCA episode collection gathered in the Dallas–Fortworth area by the DFW center for resuscitation research (UTSW, Dallas). Every episode was recorded using the Philips HeartStart MRx device, which acquires the ECG signal and the thoracic impedance through the defibrillation pads. The ECG signal was acquired with a sampling frequency of 250 Hz and a resolution of 1.03 μV per least significant bit. Additionally, some episodes included the chest compression depth signal, which in conjunction with the impedance signal, permitted identifying the intervals with chest compressions.

**Figure 1:**
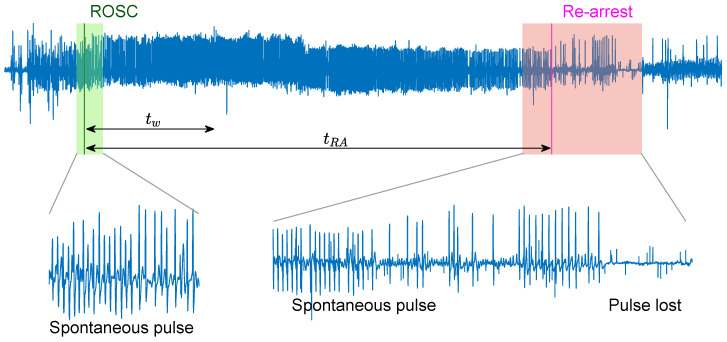
Out-of-hospital cardiac arrest (OHCA) episode where the instant of return of spontaneous circulation (ROSC), t${}_{ROSC}$(s), is associated to the pulse generating rhythm (green), and rearrest (RA) occurs t${}_{RA}$(s) later when the rhythm degenerates into a pulseless activity and asystole (red). The segment of analysis is noted with a duration of t${}_{w}$(s).

There were a total of 797 episodes with concurrent ECG and impedance signals. Episodes with ROSC were identified based on the instant of ROSC (t${}_{ROSC}$) annotated by clinicians on the scene. No rearrest episodes (NoRA) corresponded to patients with sustained ROSC according to the clinical information in the patient’s chart, and no chest compressions until the end of the episode. A minimum duration of 2 min was required for the ROSC interval. Rearrest episodes (RA) were identified if ROSC was lost in an interval of 12 min after ROSC. Patients that suffered a rearrest after 12 min from the ROSC onset were considered in the NoRA group. Figure 1 shows a RA case, where spontaneous pulse was lost t${}_{RA}$ seconds after the onset of ROSC, t${}_{ROSC}$. The final patient cohort included 162 patients, 107 NoRA, and 55 RA cases. In the NoRA cases, the median (first quartile–third quartile) duration from the onset of ROSC to the end of episode was 300 (240–874) s. In the RA cases the median duration from ROSC onset to RA was 303 (195–410) s.

## 3. Methods

The rearrest prediction algorithm proposed in this manuscript was applied to segments of $t_{w}$ minutes of ECG signal extracted right after $t_{ROSC}$, as shown in Figure 1. For case number *i* a vector of 21 features, $v_{i}=\left\{v_{i,1},\cdots ,v_{i,21}\right\}$, was computed for each segment and a machine learning classifier applied for the binary classification ($y_{i}=\left\{0,1\right\}=\left\{\mathrm{NoRA},\mathrm{RA}\right\}$). Two segment lengths were considered in the model, $t_{w}=1\;\min$ and $t_{w}=2\;\min$.

### 3.1. ECG Processing and Feature Extraction

A total of 21 features (Table 1) were extracted to vectorize the ECG segment: 17 were based on HRV metrics as proposed in [14], and four new features were incorporated based on the ECG waveform.

**Figure 2:**
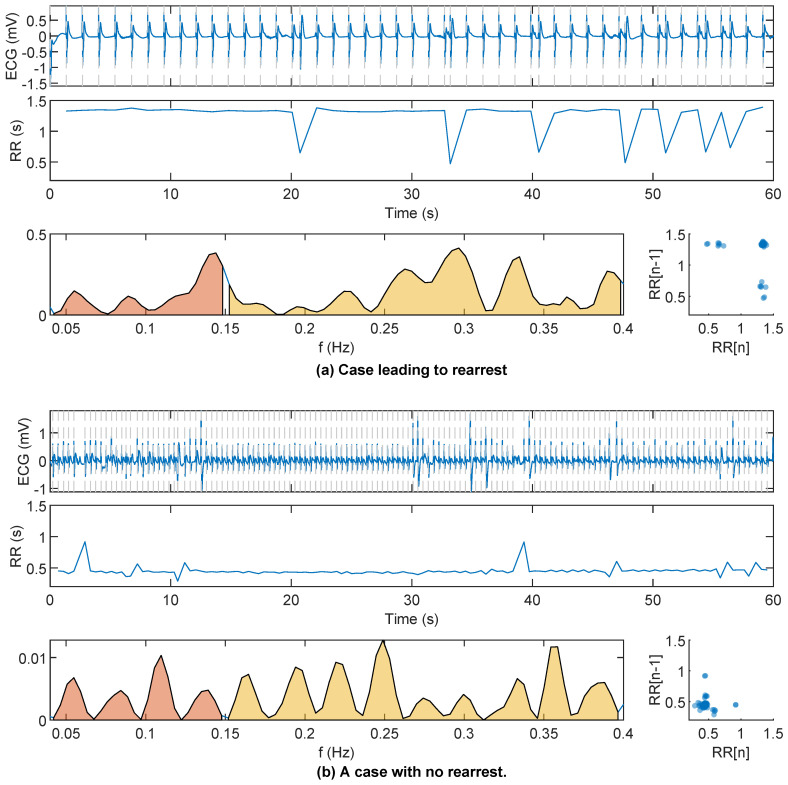
Signals corresponding to RA and no rearrest (NoRA) cases are plotted in panels (**a**,**b**), respectively. The ECG signal for $t_{w}=1\;\min$, the RR sequence, its power spectrum and and the Poincaré plot are shown.

First, the ECG signal was filtered between 0.5 and 40 Hz using order 4 Butterworth (zero-phase) filter to remove baseline wander and high frequency noise. Second, HRV features were computed using the R peaks detected using the well-known Hamilton–Tompkins QRS detector [15]. A variance-based correction was applied to prevent false negative heartbeat detections caused by large amplitude changes in the R-peaks. The impact of spiky artifacts in the adaptive thresholding of the QRS detector was thus reduced and the RR series were constructed. Examples of RR series for a RA and a NoRA case can be observed in Figure 2.

The HRV features computed using the RR series can be divided into three groups [16]:

- **Time domain features.** The classic metrics of RR variability were computed: mean RR interval ($v_{1}$), standard deviation ($v_{2}$), root mean square of the successive differences ($v_{3}$), coefficient of variation ($v_{4}=v_{2}/v_{1}$) [17], number of RR intervals that differ more than 50 ms ($v_{5}$), and the interquartile range of RR intervals ($v_{6}$).
- **Frequency domain features.** First, the spectrum of the RR sequence was computed using the Lomb–Scargle periodogram for unevenly sampled signals [18]. Then, two different frequency bands were analyzed, the low-frequency or LF band (0.04–0.15 Hz) and the high-frequency or HF band (0.15–0.4 Hz). The computed features were the absolute and relative power in the LF band ($v_{7}$ and $v_{8}$), the absolute and relative power in the HF band ($v_{9}$ and $v_{10}$), the relation between LF and HF power ($v_{11}$), and the peak frequencies in LF and HF bands ($v_{12}$ and $v_{13}$).
- **Nonlinear features.** Self similarity of the RR samples was evaluated using the Poincaré plot and entropy-based features. From the Poincaré plot the variability was measured with the width, SD1${}^{2}$, and depth, SD2${}^{2}$, of the ellipse, $v_{14}$ and $v_{15}$, respectively. Their relation was computed as $v_{16}$. The sample entropy of the RR series ($v_{17}$) was computed from a cubic interpolation of the RR series to form a uniformly sampled series (10 Hz), and m=1 and r=0.2 were used [19].

Additionally, four features were computed using the ECG signal, three of them proposed in [13] ($v_{18}$, $v_{19}$, and $v_{21}$). They were computed as follows.

- The centroid frequency, $v_{18}$, was computed based on the power spectral density (PSD) of the ECG signal. The PSD was estimated for the $f_{i}$ frequencies using Welch’s periodogram with a signal window of 12 s, an overlap of 50% and a fast Fourier transform of 4096 points:
  (1) $$v_{18}=\frac{\sum_{i}PSD\left(f_{i}\right)\;\cdot \;f_{i}}{\sum_{i}PSD\left(f_{i}\right)}$$
- The mean of the absolute values of the samples of the ECG segment, $v_{19}$.
- The relative QRS-power, as the power of the signal concentrated in the frequency band corresponding to the QRS complexes (5–14 Hz) [15,20]:
  (2) $$v_{20}=\frac{\sum_{f_{i}=5}^{f_{i}=14}PSD\left(f_{i}\right)}{\sum_{i}PSD\left(f_{i}\right)}$$
- The variability of the duration of the QRS complexes. QRS complexes were delineated using a wavelet based algorithm [21] and the standard deviation of their durations was $v_{21}$.

Table 1 provides a quick reference for the meaning of the $v_{i}$ features.

Figure 2 shows the ECG segment and the RR sequence for $t_{w}=1$ min in an RA and NoRA case. The RR instants (marked), the RR spectrum (LF and HF highlighted), and the Poincaré diagram are plotted. Larger variability of the RR series, a more disperse Poincaré plot, and more power concentration in the high frequency band were all associated to RA.

### 3.2. Building the RF Classifier

First, an univariate analysis was carried out to analyze the power of each feature to discriminate RA and NoRA cases. A cost-sensitive logistic regression classifier was fitted using a single feature in the training set and the performance metrics were obtained for that model in the test set (see Section 3.3). Then, a Random Forest (RF) classifier was used to combine all the features for several reasons: it can learn nonlinear mappings, it can be easily adapted for imbalanced datasets, and, besides allowing an embedded feature selection, feature importance can be estimated. Moreover, in our preliminary tests with other machine learning models the RF produced the best classification results. The RF classifier is an ensemble of *B* decision trees (weak learners) that produce uncorrelated predictions, and the final label is decided by majority voting [22]. Uncorrelated decisions are made by using different bootstraps of the training data to train each weak learner, and also a limited set of randomly selected features are used at each tree split. The importance of each feature can be estimated by permuting the values of each feature and looking at the increase in the out-of-bag error (error measured using the data that each weak learner did not see during the training process). The following two steps were followed.

- The RF was trained using the training dataset at hand and the importance of each feature was computed and correspondingly sorted.
- The RF was trained again using the same training data and using only the most important $N_{f}$ features from the previous ranking. Considering the class imbalance in our study (≈34/66%), the number of instances per class were balanced when creating the bootstraps to train each tree by oversampling the minority class. The RF model was evaluated with the testing dataset in hand.

Both RFs were trained using B=300 weak learners and each tree was trained using only 5% of the data. Bootstrapping was made using sampling with replacement, i.e., repeated instances were possible. For binary classification problems, the number of trees that predicted that a certain instance is positive divided by *B* can be interpreted as the probability or likelihood of the instance being positive.

### 3.3. Evaluation

The RF model was trained and tested using patient-wise and stratified 5-fold cross-validation data partition. Data were divided in five nonoverlapping groups, one was used for testing and the other four for training. This is repeated five times so every patient is used in the training and test sets. The procedure was repeated 100 times to estimate the statistical distributions of the performance metrics in terms of median (interquartile range (IQR)). The standard metrics for binary classification problems were considered.

The classification problem in this study involved two unbalanced classes: a negative class with the majority of the instances (NoRA), and a minority positive class (RA). In this scenario, two diagnostic tools are helpful to evaluate the models: receiver operating characteristics (ROC) and precision–recall (PR) curves. These curves are calculated evaluating corresponding performance metrics for different thresholds of the likelihoods given by the RF classifier. The following metrics were considered; sensitivity (Se) or recall (probability of detecting a RA case correctly), specificity (Sp, probability of detecting a NoRA case correctly), precision (probability that a positive detection corresponds to a positive case) and the harmonic mean between precision and recall ($F_{1}$ score). Areas under both curves, area under receiver operating characteristics curve (AUROC) and area under precision–recall curve (AUPRC), are good representative metrics to evaluate the performance of the model. Every metric is given as percentage.

## 4. Results

In the QRS detection, the variance based filter only changed the detections of the Hamilton-Tompkins algorithm in five cases (3% of episodes), and less than 0.3% of the samples were modified in those cases. To asses the quality of QRS detection and the RR series derived thereof, a signal quality index was adopted, the proportion of beats that are detected by two different QRS detectors over all detected beats [23]. As proposed by the authors of [23], we used a QRS detector robust to noise (Hamilton–Tompkins [15]) and a detector based on a length transform proposed by Zong et al. [24], which is more sensitive at lower signal-to-noise ratios. Median (first quartile–third quartile) agreement between the QRS detectors was 98.4% (90.7–99.6%), showing the good quality of the data.

The analysis of the logistic regression classifier for single features yielded median AUROC and AUPRC values for $t_{w}=1\;\min$ in the range of 52.0 to 65.1 and 29.3 to 50.3, respectively. Similar results were obtained for $t_{w}=2$ min, with AUROC in the range of 53.7 to 66.2 and AUPRC in the range of 28.2 to 50.1. A random classifier in this case would correspond to AUROC=50.0 and AUPRC=34.0. Table 2 shows the distributions and median AUROC/AUPRC for the top 10 features (highest harmonic mean between AUROC and AUPRC) for $t_{w}=1$ min and $t_{w}=2$ min, respectively. It can be observed that time features like $v_{2}$ and $v_{4}$ were important for both values of $t_{w}$, showing that the variability of the RR sequence is a powerful discriminative feature. Nonlinear features measured through Poincaré plots and entropy ($v_{15}$ and $v_{17}$) also showed high AUROC with medians of 65.0 and 65.5, respectively.

The correlation analysis between the features, based on the Pearson coefficient, $r^{2}$, showed high correlation between features in the same or different domains. Thus, $v_{2}$ showed good correlation ($r^{2}>0.75$) with $v_{3}$, $v_{4}$, $v_{14}$ and $v_{15}$, and also $v_{4}$ with $v_{14}$.

The median (IQR) values of the features showed that RA patients presented more variable RR intervals, reflected in higher values of time HRV features, $v_{2}$ and $v_{4}$, and in a wider Poincaré plot as measured by $v_{15}$. The entropy of the RR series ($v_{17}$) was lower in RA cases, suggesting a more regular/predictable time series.

**Figure 3:**
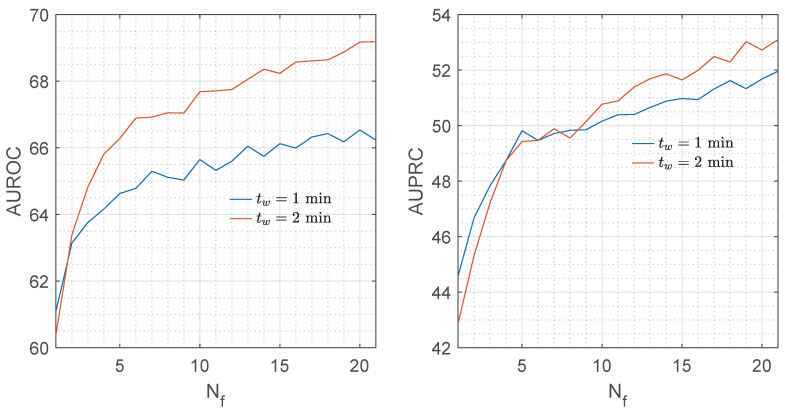
AUROC and AUPRC for the random forest (RF) classifier in function of the number of features of the model, $N_{f}$, for $t_{w}=1$ min and $t_{w}=2$ min.

Figure 3 shows the median AUROC and AUPRC for the RF classifier as a function of the number of features considered in the model, $N_{f}$. Adding features to the model improved both metrics.

**Figure 4:**
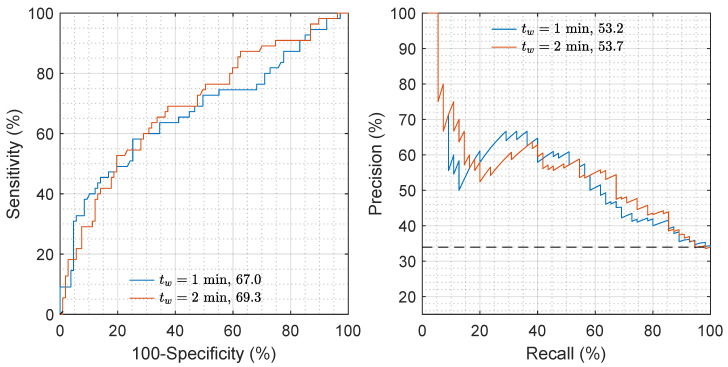
Receiver operating characteristics (ROC) and precision–recall (PR) curves for both values of $t_{w}$. The repetition that was closest to the median AUROC or AUPRC was chosen to depict the curves. The AUROC and AUPRC increased from 67.0 to 69.3, and from 53.2 to 53.7, respectively, when tw=2 min were considered.

**Figure 5:**
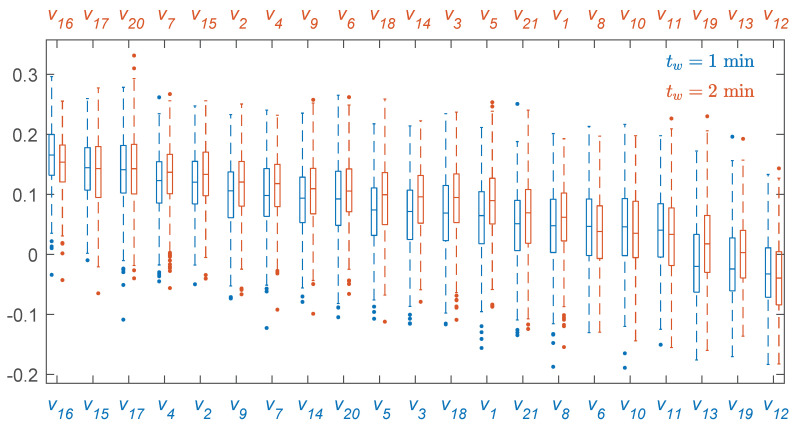
Distributions of feature importances given by the RF classifier sorted by importance for $t_{w}=1$ min (blue) and $t_{w}=2$ min (red).

Figure 4 shows the ROC and PR curves for the repetition closest to the median performance. The AUROC and AUPRC increased a median of 2 and 1 points for $t_{w}=2$ min, showing that longer intervals improved the accuracy of the features in general and that of the spectral features in particular. The distributions of importance for each feature, depicted in Figure 5, show that most of the features had a positive importance and were relevant for the RF model. Features like $v_{20}$, $v_{17}$, $v_{7}$, $v_{15}$, or $v_{2}$ were in the top 10 when analyzed individually (see Table 2). Others, like $v_{16}$, were relevant when combined with the rest in the predictive model and when considering a RF classifier instead of a cost-sensitive logistic regression classifier.

Table 3 shows the overall metrics for the RF classifier for the thresholds that maximized the $F_{1}$ score. Adding the ECG features, $v_{18}$–$v_{21}$, to the HRV features significantly increased Se for both $t_{w}$ values (p<0.05 according to the Mann–Whitney test). For $t_{w}=2$ min the AUROC and AUPRC increased 2 points, and the Se almost 6 points, meaning that 20% of the missclassified RA cases would be correctly detected.

## 5. Discussion

The final objective of prehospital treatment of OHCA is to recover spontaneous pulse. However, many detrimental factors may induce a secondary cardiac arrest, or rearrest, before arrival to hospital. These rearrest events reduce the probability of survival to hospital discharge [7,8,25,26,27]. Currently, clinicians apply expert knowledge on scene to foresee if a patient who has achieved ROSC should be transported immediately, or if the patient requires longer on-site treatment. Defibrillators show physiologic signals on screen but do not provide tools to assist clinicians on the prediction of a secondary arrest. To the best of our knowledge, this study provides the first automated method based on the ECG to predict rearrest. This is important because the ECG is routinely recorded in all defibrillators. The method is based on a RF classifier using HRV features and ECG waveform features, and showed a Se and Sp of 67%.

Fluctuating heart rates are frequent in organized rhythms during cardiac arrest. When spontaneous circulation is restored the QRS complexes may still be irregular in morphology and rate. ECG features associated to the heart rate have been used to successfully predict time to RA [12], especially the standard deviation of the measured heart rate. The standard deviation of the RR intervals ($v_{2}$) is a similar measure, and was also one of the most important features of the RF classifier. Moreover, $v_{2}$ alone showed a median AUROC of 66.2 (65.3–67.0) and median AUPPRC of 50.1 (49.3–50.7).

HRV features have been widely used in non-arrest situations to detect and predict cardiac arrhythmias [20,28,29]. They were originally designed to analyze long intervals, minutes, or even hours, in hemodynamically stable patients. Interestingly, in this study, HRV features have been proven to be good predictors of RA even with segments as short as $t_{w}=1\;\min$. The spectral HRV features showed better performance for $t_{w}=2\;\min$ due the better resolution of the RR spectrum associated to longer analysis segments. We observed median increases of 1–2 points in the AUROC when the segment was increased from $t_{w}=1\;\min$ to $t_{w}=2\;\min$.

In a post-cardiac arrest scenario, the patient may not be breathing spontaneously after ROSC, and rescuers should artificially ventilate the patient. This may cause reduced respiratory-related heart rate dynamics and may influence HRV features. However, more studies are needed to analyze the relationship between the HRV metrics and ventilation metrics of the patient.

During treatment of OHCA patients many rhythm transitions occur, such as from an initial ventricular fibrillation to recovery of spontaneous pulse. Many studies have focused on the analysis of the prevalence and the prediction of rhythm transitions [30,31], including the transition from ROSC to another cardiac rhythm, that is, rearrest. A short time predictor was proposed in [13] using features $v_{18}$, $v_{19}$, and $v_{21}$ to predict the rhythm in the next 3 s with an AUROC of 73. Our clinical context was different as we developed a model for patients that recovered a stable spontaneous pulse, and applied our model to predict a rearrest occurring on average 5 (6–7) min later. This is a much more challenging scenario, but of great clinical importance as it would allow clinicians to make more informed decision on transport to hospital after spontaneous pulse is recovered.

In this study, we also confirmed that the ECG waveform features significantly improved the performance of the RF model. Compared to the RF based exclusively on HRV features that we proposed in [14], the combination of HRV and ECG features improved the AUROC and AUPRC in 2 points when we increased the number of patients in the dataset by 65%. These are the first results of a machine learning solution to predict rearrest, and the RF model showed that including more features increased the accuracy of the method. The prediction of rearrest is a clinically important topic in OHCA treatment, and our results show that it is a challenging one. In the future, more sources of information available during treatment could be added to the models, including measures of the respiratory function (capnogram), cerebral state (cerebral oximetry or EEG-based bispectral analysis), or even blood pressure. These signals are not universal in OHCA treatment, but could be used to provide complementary information to that derived from HRV/ECG analysis.

The duration of the analysis interval of the ECG is important to predict rearrest. Our results showed that the performance improved when longer analysis windows were used, with differences of 2 points in AUROC when the duration of the analysis window was increased from 1 min to 2 min. In order to confirm that hypothesis, we replicated the analysis using the typical short window for HRV parameter calculations ($t_{w}=5\;\min$). This reduced the sample size to 98 (26 RA and 72 NoRA). In this subset the AUC when $t_{w}=5\;\min$ was 8 points larger than for $t_{w}=2\;\min$. This shows that longer analysis intervals improve the accuracy for the prediction of rearrest. However, in an OHCA scenario, time to clinical decisions and interventions is critical for survival, so a trade-off must be found between the detection of critical situations (rearrest) and the time needed to identify those situations. Longer delays to transport the patient in sustained ROSC to a hospital for a percutaneous coronary intervention may substantially lower the probability of survival of the patient [32]. Consequently, short analysis intervals (if sufficient for a diagnosis) should always be adopted.

This study has several limitations. The first one is the small patient cohort (162 cases), which, albeit being the largest cohort studied to date for this purpose, is still insufficient to draw conclusive results. Further studies on larger cohorts are needed, based on the evidence provided in this study. Second, the interpretation of the HRV parameters in a cardiac arrest context is controversial. Our results show they convey important information on the prediction of rearrest, but their physiological interpretation as measures of how cardiac arrest affects the autonomic nervous system are unclear. Third, in OHCA, time constraints in clinical interventions are of life and death importance; this limits the time available for the decisions and thus the segment lengths to compute HRV metrics. Short segments under 5-min should be used to compute HRV metrics, which further complicates the accuracy and interpretability of these measures. Finally, the conditions in which the ECG is recorded in a prehospital setting (hygiene, electrode contact, movement, and interventions) make QRS detection and thus RR-series calculations more challenging than in controlled hospital or laboratory conditions.

## 6. Conclusions

A RF model to predict a secondary arrest in the out-of-hospital setting is proposed using only 1 or 2 min of ECG signal right after return of spontaneous circulation. This manuscript shows that ECG signal and HRV metrics contain information about rearrest events, further studies are needed to confirm and improve our results.

## Figures and Tables

**Table 1:** Overview of the computed features.

**Time-domain HRV features**	**Non-linear HRV features**
$v_{1}:$ Mean RR	$v_{14}:$ SD1${}^{2}$
$v_{2}:$ Standard deviation RR	$v_{15}:$ SD2${}^{2}$
$v_{3}:$ RMSSD	$v_{16}:$ SD1${}^{2}$/SD2${}^{2}$
$v_{4}:$ Coefficient of variation	$v_{17}:$ Sample entropy
$v_{5}:$ nNN50	**Signal-level features**
$v_{6}:$ Interquartile range RR	$v_{18}:$ Centroid frequency
**Frequency-domain HRV features**	$v_{19}:$ Signal amplitude
$v_{7}:$ LF absolute power	$v_{20}:$ Relative QRS power
$v_{8}:$ LF relative power	$v_{21}:$ Standard deviation of QRS width
$v_{9}:$ HF absolute power	
$v_{10}:$ HF relative power	
$v_{11}:$ LF/HF power	
$v_{12}:$ LF peak frequency	
$v_{13}:$ HF peak frequency	

**Table 2:** Distributions of the values for the top 10 features, represented as median (IQR) for each class, and their median area under receiver operating characteristics curve (AUROC) and area under precision–recall curve (AUPRC) values. Results for $t_{w}=1$ min and $t_{w}=2$ min are shown.

$t_{w}=1$ min						$t_{w}=2$ min				
Feature	NoRA	RA	AUROC	AUPRC		Feature	NoRA	RA	AUROC	AUPRC
$v_{15}$	0.01 (0.02)	0.03 (0.10)	65.0	50.3		$v_{2}$	0.08 (0.12)	0.21 (0.37)	66.2	50.1
$v_{2}$	0.07 (0.10)	0.15 (0.25)	64.9	50.2		$v_{4}$	0.16 (0.19)	0.29 (0.40)	65.7	49.4
$v_{7}$	0.00 (0.00)	0.00 (0.01)	63.3	49.4		$v_{6}$	0.06 (0.11)	0.14 (0.26)	63.4	48.7
$v_{4}$	0.14 (0.17)	0.23 (0.24)	64.2	48.9		$v_{17}$	0.31 (0.45)	0.18 (0.27)	65.5	47.4
$v_{9}$	0.00 (0.00)	0.01 (0.03)	62.4	47.8		$v_{3}$	0.57 (0.23)	0.71 (0.49)	63.3	48.4
$v_{14}$	0.05 (0.06)	0.09 (0.18)	61.9	47.8		$v_{14}$	0.05 (0.09)	0.11 (0.20)	64.0	47.7
$v_{17}$	0.35 (0.51)	0.20 (0.30)	65.1	45.9		$v_{15}$	0.01 (0.02)	0.05 (0.22)	61.7	47.0
$v_{3}$	0.56 (0.26)	0.68 (0.45)	60.3	48.2		$v_{20}$	0.38 (0.20)	0.28 (0.22)	64.5	45.4
$v_{1}$	0.55 (0.24)	0.63 (0.38)	59.3	46.8		$v_{5}$	216 (81)	180 (104)	61.6	46.6
$v_{5}$	106 (45)	93 (54)	59.4	45.4		$v_{7}$	0.00 (0.00)	0.01 (0.03)	60.7	46.4

**Table 3:** Performance metrics for the RF model in median (IQR) using only the HRV features and using all the features for both interval analyses, $t_{w}=1$ min and $t_{w}=2$ min.

	$t_{w}$	Se or Recall (%)	Sp (%)	Precision (%)	$F_{1}$ (%)	AUROC	AUPRC
HRV features	1 min	57.3 (11.8)	75.7 (14.5)	54.5 (9.8)	55.8 (2.8)	65.4 (2.3)	51.2 (2.9)
	2 min	61.8 (6.4)	72.9 (6.1)	54.4 (4.6)	57.6 (2.0)	67.3 (2.0)	50.7 (2.7)
All features	1 min	63.6 (15.5)	69.2 (20.6)	51.5 (10.0)	55.4 (3.1)	66.2 (2.2)	52.0 (2.6)
	2 min	67.3 (9.1)	67.3 (10.3)	51.4 (5.3)	57.9 (1.7)	69.2 (1.6)	53.1 (3.0)

## Abbreviations

The following abbreviations are used in this manuscript:

AUROC	area under ROC curve
AUPRC	area under PR curve
CPR	cardiopulmonary resuscitation
ECG	electrocardiogram
HF	high frequency
HRV	heart rate variability
LF	low frequency
NoRA	no rearrest
OHCA	out-of-hospital cardiac arrest
PR	precision–recall
RA	rearrest
RF	random forest
ROC	receiver operating characteristics
ROSC	return of spontaneous circulation
